# Geopolymer Materials from Fly Ash—A Sustainable Approach to Hazardous Waste Management

**DOI:** 10.3390/ma17143515

**Published:** 2024-07-16

**Authors:** Katarzyna Zarębska, Jakub Szczurowski, Joanna Muszyńska, Paweł Baran

**Affiliations:** 1 Faculty of Environmental Engineering, Geomatics and Renewable Energy, Kielce University of Technology, 25-314 Kielce, Poland; jdlugosz@tu.kielce.pl; 2 Faculty of Energy and Fuels, AGH University of Cracow, Mickiewicza 30, 30-059 Kraków, Poland; szczurow@agh.edu.pl (J.S.); baranp@agh.edu.pl (P.B.)

**Keywords:** fly ash, geopolymer, heavy metal leaching, municipal waste incineration, sustainable waste management

## Abstract

This study explores the utilisation challenges of fly ash from municipal waste incineration, specifically focusing on ash from a dry desulphurisation plant (DDS), which is categorised as hazardous due to its high heavy metal content. The ash’s low silicon and calcium contents restrict its standalone utility. Laboratory investigations initially revealed that geopolymers derived solely from fly ash after flue gas treatment (FGT), in combination with coal combustion fly ash, exhibited low compressive strength (below 0.6 MPa). However, the study demonstrated significant improvements by modifying the FGT ash through water leaching. This process enhanced its performance when mixed with high-silica and -aluminium fly ash, resulting in geopolymers achieving compressive strengths of up to 18 MPa. Comparable strength outcomes were observed when the modified ash was blended with commercial cement. Leachability tests conducted for heavy metals (HMs) such as copper, zinc, lead, cadmium, and nickel indicated that their concentrations fell below the regulatory limits for landfill disposal: 2, 4, 0.5, 0.04, and 0.4 mg/kg, respectively. These results underscore the effectiveness of water-washing FGT ash in conjunction with other materials for producing geopolymers, contributing to sustainable waste management practices.

## 1. Introduction

Municipal solid waste (MSW) incineration is an established method of waste treatment with simultaneous energy recovery, firmly embedded in the circular economy framework, which aims to minimise waste and maximise resource recovery [1]. However, this process generates byproducts, such as fly ash, which is classified as hazardous waste under EU Directive 91/689/EEC [2]. The EU and its member states, including Poland, require strict waste management practices, emphasising waste prevention and recovery in accordance with the waste hierarchy [3]. To convert fly ash into a safe material suitable for landfilling or reuse, it is necessary to reduce the leaching of potentially toxic elements. Various methods are being considered, among which stabilisation/solidification is often mentioned.

The solidification/stabilisation method of hazardous waste disposal was developed in the 1960s to neutralise radioactive waste [4]. Over time, it has been adapted to the disposal of other wastes—in particular, industrial and hazardous wastes or sewage sludge. The solidification process makes it possible to change the physical and chemical properties of the waste and to reduce the solubility and leachability of hazardous compounds [5,6]. The addition of incineration byproducts, such as fly ash and granulated blast furnace slag, as main components in cement composition is currently practiced by many producers and aligns with the concept of sustainable management of raw materials and waste. Solidification/stabilisation is the most common technique for neutralising and stabilising fly ash [7]. The release rate of heavy metals can be effectively controlled using cement or polymeric binders [8]. This method limits the emission of heavy metals through physical and chemical interactions, leading to encapsulation, fixation, or sorption of pollutants [9]. The increasingly common production of composites containing waste, often hazardous, requires an assessment of the release of hazardous substances (especially heavy metals) from these materials. Mineral binders are used to create matrices that solidify hazardous waste containing heavy metals. The system for assessing the release of hazardous substances (especially heavy metals) from mineral composites is particularly relevant, as these composites are more commonly used in sustainable waste management practices. The amount of substances released from construction materials into the environment is determined by many factors, including the form of the construction material (monolith or crushed material) and environmental factors such as soil, water, sewage, varying temperature and humidity, and chemical environmental conditions.

Direct technologies for the removal of hazardous pollutants from municipal solid waste incineration ash have also been proposed. Increasingly, research in the field of fly ash treatment focuses on thermal, leaching, extraction, and solidification or chemical stabilisation methods [5,10,11,12]. The use of water as an extraction medium is considered to be the simplest separation method, effectively removing soluble salts. However, the use of selected inorganic acids for metal extraction, while effective, significantly changes the pH and properties of the ash [13] and generates effluent, which negatively affects the economics of the process [9,14]. Thermal processes such as smelting, vitrification, or sintering yield better results than extraction processes by reducing the leachability of heavy metals [15,16]. Exposure to high temperatures leads to the decomposition of hazardous substances, with some heavy metals evaporating and others being encapsulated in the vitrified mass. However, these methods have drawbacks, including the release of heavy metals into the atmosphere and significant energy consumption [14,16].

The potential contamination and release of heavy metals from fly ash are typically assessed using leaching tests to determine the leachability of contaminants. In the European market, the most commonly used leachability tests are the USEPA Toxicity Characteristic Leaching Procedure (TCLP) and the European Standard Test) [17,18,19]. In EN 12457/1-4, the extraction medium is distilled water with a liquid-to-solid (L/S) ratio of 10 for 24 h. In contrast, the USEPA TCLP method involves continuous stirring of the granular material in an acetic acid solution with an L/S ratio of 20 for 18 h [20]. Inductively coupled plasma optical emission spectrometry (ICP-OES) and atomic fluorescence spectrometry (AFS) are two standard methods used to analyse heavy metals in fly ash [21].

The management and use of fly ash remain critical challenges in waste management. Various methods are being investigated to process and incorporate fly ash into building materials, aiming to avoid the high costs associated with hazardous waste disposal [22,23,24,25,26,27,28]. Additionally, research has explored the potential for reclaiming degraded and post-development sites, including municipal landfills, by utilising selected municipal wastes such as incinerator ash and stabilised sewage sludge. Results indicate that mineral–organic composites produced from these wastes contain low levels of water-soluble heavy metals, making them suitable for reclamation purposes [29].

The leachability of heavy metals is significantly enhanced under highly acidic conditions, but their release rate can be minimised if the pH is maintained within the range of 5–12. In addition to extrinsic conditions, fly ash properties such as CaO content, soluble chlorine (S-Cl) content, and pH can significantly affect the leachability of heavy metals. Higher CaO and lower soluble chlorine contents efficiently reduce the leachability of fly ash and remove toxic heavy metals; however, combining water washing with other methods can be a practical strategy for fly ash stabilisation. Pre-washing of fly ash can improve the efficiency of heavy metal removal by acid extraction, by reducing the alkaline nature of the fly ash. Additionally, water washing after acid extraction shows promise for stabilising heavy metals, specifically Pb [17].

Therefore, combinations such as water washing/solidification, water washing/acid leaching, water washing/electrodialytic separation, water washing/thermal treatment, and water washing/chemical stabilisation/thermal treatment are proposed as effective approaches for the highly efficient removal and stabilisation of HMs, considering economic and environmental factors.

In a previous study, Wang et al. proposed using the geopolymerisation process as a viable method for disposing of municipal solid waste incineration fly ash (MSWIFA). This process offers the advantage of immobilising heavy metals and harnessing the pozzolanic properties of the ash to produce eco-friendly cementitious materials. In their research, coal fly ash (FA) and metakaolin (MK) were utilised to create a geopolymer composite, where MK was partially replaced by varying proportions of MSWIFA using the alkaline activation method. The leaching levels of Cd and Pb in the raw material MSWIFA exceeded the standard limits, while the leaching levels for every heavy metal ion in the geopolymer paste with 40% MK replaced by MSWIFA were lower than the Chinese standards [30].

Another study [31] investigated the potential of using the geopolymerisation process to immobilise ash from municipal solid waste incinerators. The waste was solidified in geopolymer matrices made from (i) fly ash from the combustion of bituminous coal or (ii) metakaolin. The study demonstrated that the geopolymerisation process effectively immobilises compounds and elements such as chlorides, sulphates, fluorides, barium, and zinc. Importantly, the results indicated that the level of immobilisation was significantly higher in metakaolin-based geopolymers compared to those made from coal fly ash.

Lu et al. [32] investigated the feasibility of partially replacing pulverised fly ash (PFA) with MSWIFA to produce ambient cured geopolymers. They examined the influence of mixing parameters on the compressive strength of the geopolymer paste. Microstructural analysis revealed that the main reaction product was a combination of calcium silicate hydrate gel and aluminosilicate gel. The concentration of leachable heavy metals in MSWIFA blended PFA geopolymer mortar significantly plummeted to less than 1%. For all curing days, including 7 and 28 days, the concentrations of the analysed metals were within the limitations presented in the relevant standards.

The issue of the cost associated with incinerating municipal waste is widely discussed, with thermal waste treatment technology having both supporters and opponents. Analysing the costs of incineration is particularly intriguing when contrasted with the strong emphasis on waste recycling. Currently, the predominant methods for waste disposal are landfilling and incineration. However, landfilling is increasingly unpopular due to its noticeable carbon footprint and other environmental impacts, such as groundwater contamination, soil erosion, and land use. In comparison, incineration reduces the waste volume by 90% and mass by 75% [16], while also generating significant amounts of energy in the forms of heat and electricity [9]. The US Environmental Protection Agency is giving priority to waste reduction strategies, including source reduction [33]. Given the vast expanse of the United States, space constraints are generally not a significant factor encouraging the construction of municipal waste incinerators. Landfilling is often seen as a more cost-effective option in the short term due to lower initial construction costs compared to waste incineration plants. In contrast, regions with dense populations and limited space, such as many European countries and Japan, favour incineration with energy recovery due to space limitations. Various approaches, including small waste-to-energy plants, large-scale waste-to-energy facilities with complex logistics, and mechanical–biological treatment before incineration, have been proposed and compared to strategies where mixed municipal waste is primarily landfilled. Studies [34] have assessed the trade-offs between economically viable and environmentally acceptable solutions, suggesting potential reductions in greenhouse gas emissions of nearly 150% with marginal increases in waste treatment costs (approximately EUR 2.5/t). China, with its diverse urbanisation challenges, views waste incineration as a crucial component of its “zero-waste city” strategy [35].

This study focuses on the solidification and stabilisation of fly ash for use in construction materials, promoting sustainable waste management practices. It investigates methods to process fly ash to avoid the costly disposal of hazardous waste. In addition to researching the synthesis of geopolymeric materials with low leachability of heavy metals, this study proposes direct technologies for removing hazardous contaminants. The primary objective is to develop a management method for FGT ash that meets the stringent environmental regulations of the EU and contributes to the sustainability goals of the circular economy.

The objective was achieved by developing a method for managing MSW incinerator ash after desulphurisation, aimed at producing samples with sufficient compressive strength and minimal leaching of heavy metals. Based on their literature review, the authors propose synthesising a geopolymer material using FGT ash treated with water and specific additives. They also suggest alkaline activation of cementitious components incorporated into the synthesis process. Selected samples underwent leachability tests for zinc, lead, copper, nickel, and cadmium to assess the effectiveness of the method.

## 2. Methods and Materials’ Characterisation

### 2.1. Leachability Analysis of Heavy Metals

The leachability of selected heavy metals from FGT ash after washing was assessed using advanced inductively coupled plasma optical emission spectrometry (ICP-OES) in accordance with PN-EN ISO 11885:2009 [36]. This analysis was complemented by sequential extraction procedures to determine the speciation of heavy metals and their potential mobility under varying environmental conditions. Leaching tests were conducted to simulate different pH levels and redox conditions, aiming to mimic natural environmental scenarios.

The results obtained provide insights into the long-term stability of encapsulated heavy metals within FGT ash and aid in evaluating the environmental risks associated with their potential release.

### 2.2. Analysis of Ash and Leachate Following Water Extraction 

Following the water extraction process, the FGT ash and resulting leachates underwent a comprehensive analysis using the S-METAXHB1 and S-METAXHB2 methods with ICP-OES (Agilent 5100 SVDV, Santa Clara, CA, USA) at the ALS laboratory in the Czech Republic. This involved a detailed assessment of the soluble fractions of heavy metals, offering insights into the immediate environmental impact of the leachate.

Additionally, the post-extraction characterisation of the ash focused on identifying changes in mineralogy, the stability of remaining heavy metals, and alterations in physical properties. These analyses are crucial for developing further treatment strategies to immobilise metals within the ash matrix, and for assessing the suitability of the processed ash for various applications in accordance with the principles of waste recovery and reuse.

### 2.3. Compressive Strength of Alkali-Activated Materials

The assessment of the compressive strength of alkali-activated materials derived from FGT ash was performed using an automated press (made by Multiserw, Brzeźnica, Poland), in accordance with the PN-EN196-1:2016-07 standard [37]. This method involves testing cubic specimens measuring 40 × 40 × 40 mm^3^ to determine their strength after curing for 7 and 28 days.

To enhance the accuracy of the analysis, the experimental setup included controlled curing conditions designed to simulate real-world application environments. This approach ensures that the measured compressive strength values reflect the performance of the materials under practical conditions.

The results aim to offer a thorough understanding of the mechanical performance and potential structural applications of these geopolymers within the construction industry. Each reported result represents the average of duplicate experiments, ensuring the reproducibility and reliability of the findings. This approach enhances confidence in the accuracy of the data and their applicability for practical use in construction materials.

## 3. Materials

The fly ash (FGT) used in this study was obtained from a municipal waste incineration plant in Poland. FGT residues are generated as byproducts during desulphurisation processes in waste incineration or coal-burning power plants, where sulphur oxides react with lime in the scrubbing solution, forming insoluble calcium sulphite and calcium sulphate solids.

In addition to FGT ash, the geopolymeric samples included fly ashes derived from the combustion of bituminous coal (fly ashes K and L) and lignite (fly ash B). For some of the syntheses, Gorazdze (Poland) CEM I 42.5 R cement was used. CEM I 42.5 R is known for its rapid strength development, achieving high early and final strengths. This cement type exhibits a significant heat of hydration during hardening, reflecting its strong performance characteristics in construction applications.

## 4. Synthesis

**Figure 1:**
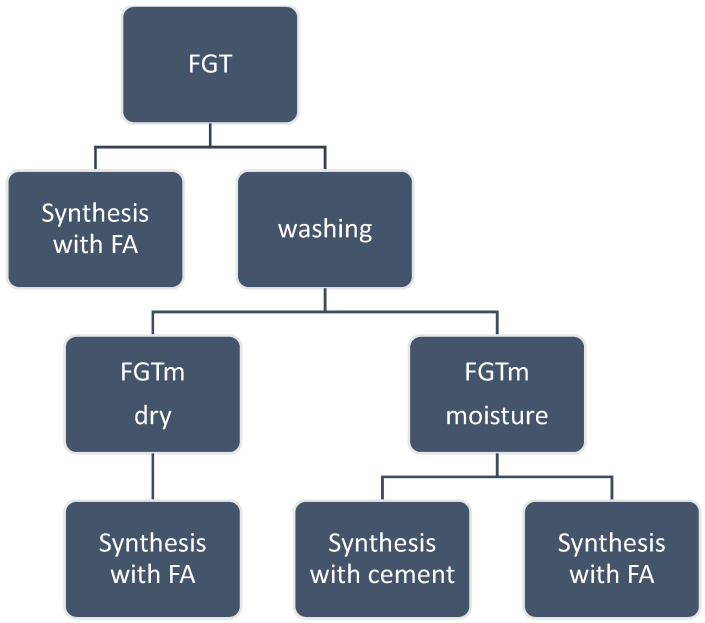
Concept of the research procedure for geopolymer synthesis.

The authors proposed a comprehensive research procedure for synthesising geopolymer samples, as illustrated in Figure 1.

In the initial phase of the research, raw FGT ash was combined with various types of fly ash (identified as B, K, and L) in the first three synthesis experiments. This phase aimed to explore the interactions and properties that arise from combining different types of fly ash with FGT ash.

For the subsequent experiments, the raw FGT ash underwent a washing process aimed at altering its physical and chemical properties. The process began by transferring the raw ash into a mixer. Distilled water was then added at a specific ratio of 30 g of ash to 100 g of water.

The mixture was continuously stirred for 2 h. This extended stirring period was crucial to ensure thorough interaction between the ash particles and the water. The consistent agitation facilitated the removal of impurities and solubilisation of some components from the ash particles.

After the 2 h stirring process, the next step involved removing the excess water from the mixture. This was achieved by filtering the mixture under reduced pressure at room temperature. The result of this filtering process was a wet form of FGT ash, referred to as FGTm. The still-wet FGTm ash was subsequently used in further syntheses, focusing on two distinct pathways.

In the first pathway, termed FGTm Moisture, the moist FGTm ash was directly utilised in synthesis along with various fly ashes and cement. The presence of moisture in the ash was expected to enhance chemical reactions and bonding during the geopolymerisation process, potentially improving the material properties.

In contrast, the second pathway, known as FGTm Dry, involved drying the FGTm ash before its use in synthesis. The dried FGTm ash was then combined with different types of fly ashes for synthesis, allowing the researchers to assess how varying moisture levels influence the final material properties.

These pathways aimed to provide insights into optimising the geopolymer synthesis process and tailoring material characteristics based on the moisture content in FGT ash.

Table 1 provides a comprehensive overview of the sample preparation for the synthesis experiments. It details the quantities of both raw and treated FGT ash, different types of fly ashes (such as B, K, and L), cement, and chemical additives utilised in each experiment. This table serves to outline the specific proportions and combinations of materials employed to investigate various formulations during the geopolymer synthesis process. It is essential for understanding the experimental setup and the variables tested for achieving the desired material properties.

To activate the materials, a liquid alkali activator was prepared by blending sodium silicate with a 10 mol/dm^3^ sodium hydroxide solution, which was mixed 2 h before the synthesis.

The preparation of the specimens began with an initial 3 min mixing of the ashes. After this initial mixing period, the alkali solution was added to the mixture, and the mixing process continued for another 3 min to ensure thorough incorporation of the solution into the ashes.

For some samples, water adjustments were necessary to achieve the correct consistency of the mortar. The goal was to obtain a homogeneous and workable mortar mixture that would set properly during the curing process.

Once the desired consistency was achieved, the fresh mortars were cast into moulds with dimensions of 40 × 40 × 160 mm. These moulds were then placed in a laboratory dryer set to a temperature of 60 °C. This pre-curing phase lasted for 24 h, during which the initial setting of the mortar took place under controlled temperature conditions.

After the 24 h pre-curing period, the samples were removed from the dryer. The next step was an extended curing period to ensure the development of the desired mechanical properties. The samples were allowed to cure for an additional 28 days. This extended curing process was crucial for the strength and durability of the final material, as it allowed for the continued chemical reactions necessary for the material to achieve its full potential. The overarching goal of the research process was to develop effective methods for managing FGT ash through its utilisation in geopolymer synthesis. The research cycle was structured to investigate how the extraction process affects the properties of the resulting materials. Given the initial low silicon and aluminium contents in raw FGT ash, each synthesis experiment incorporated additional fly ash from coal combustion, specifically lignite ash (B ash) and bituminous coal ash (K and L ashes).

Following synthesis and the determination of compressive strength, selected samples underwent analysis to evaluate the leaching potential of specific heavy metals. This assessment was crucial to ensure the environmental safety and suitability of the geopolymer materials for potential applications. By examining these aspects, this study aimed to contribute towards sustainable waste management practices while exploring new avenues for utilising industrial byproducts in construction materials.

## 5. Results and Discussion

The chemical compositions of the coal fly ashes (B, K, and L) were determined using X-ray fluorescence (XRF) analysis. The outcomes of these analyses are detailed and compared in Table 2, providing a comprehensive overview that aids in understanding their elemental compositions and potential reactivity in geopolymer synthesis.

B fly ash was distinguished by its higher calcium oxide (CaO) content of 15.12%, which can influence the setting characteristics of geopolymer materials. In contrast, K fly ash exhibited a higher concentration of silicon dioxide (SiO_2_), at 47.50%, a key component contributing to the strength and durability of geopolymers. L fly ash, on the other hand, contained a relatively elevated iron oxide (Fe_2_O_3_) content of 10.09%, impacting the colour and possibly the mechanical properties of the synthesised materials.

These variations in chemical composition are pivotal for understanding how each type of fly ash behaves during geopolymerisation. Higher SiO_2_ levels typically correlate with stronger and more resilient geopolymer matrices, while differences in CaO and Fe_2_O_3_ concentrations can affect the curing processes and overall mechanical characteristics of the resulting materials. This nuanced understanding guides the selection and formulation of fly ash combinations to optimise geopolymer synthesis for desired material properties and performance.

Calcium is the predominant element found in fly ash from municipal waste incineration, primarily originating from lime added as an alkaline reagent in the flue gas desulphurisation process [38,39]. In fly ash, calcium commonly exists in various compounds, such as calcium hydroxychloride (CaClOH), calcite (CaCO_3_), portlandite (Ca(OH)_2_), anhydrite (CaSO_4_), halite (NaCl), or syngenite (K_2_Ca(SO_4_)_2_·H_2_O) [40,41].

A significant issue with municipal waste incineration ash is the presence of trace metals like lead (Pb), zinc (Zn), barium (Ba), antimony (Sb), copper (Cu), chromium (Cr), and others, which are classified as hazardous. These metals must be carefully managed when disposing of waste in landfills to comply with European regulations. Table 3 illustrates the substantial variability in the concentrations of these metals in municipal waste incineration fly ash.

The concentrations of heavy metals in ash from municipal waste combustion are influenced by the conditions of the incineration process. Higher temperatures during combustion cause heavy metals, particularly zinc, cadmium, lead, and arsenic, to volatilise and subsequently condense onto fly ash particles carried by flue gases [47,48].

The significant variability in heavy metal contents, as shown in Table 3, can be attributed not only to the specific conditions under which the incineration process occurs but also to the diverse composition of municipal waste subjected to incineration. Municipal waste’s composition can vary widely, impacting the types and amounts of heavy metals present in the resulting ash.

### 5.1. Results of Strength Tests

The proposed research cycle aimed to develop a methodology for managing FGT ash to produce a structural material with robust strength properties and minimal leaching of heavy metals. However, due to FGT ash’s low aluminium and silicon contents, it cannot function effectively as a standalone material for geopolymer production. Nevertheless, its high calcium oxide content allows for potential blending with low-calcium F-type fly ashes. Our research indicated that directly incorporating FGT ash with coal combustion fly ashes did not yield satisfactory compressive strength results. Based on findings from the literature, the simplest method explored was leaching soluble compounds from FGT ash using water. Two approaches were tested: using ash immediately after water leaching under reduced pressure (moist ash), and using ash that underwent drying after leaching. Unfortunately, strength tests revealed that doping raw FGT ash did not improve the compressive strength (see Table 4). The results remained relatively low, with no significant increase observed even after 28 days of curing. This outcome highlights the challenges in enhancing the properties of FGT ash for geopolymer applications through straightforward leaching processes alone.

The synthesis conducted using ash after the water-washing process also failed to produce materials with sufficiently high strength. Moreover, the lack of strength increase after 28 days suggests an insufficient concentration of NaOH, which was diluted with water remaining from the leaching process. This hypothesis is supported by the outcomes of syntheses using dried ash after washing, which demonstrated a significant improvement in strength results. The results clearly indicate that an adequate concentration of the activating solution (NaOH) is crucial for dissolving the active metals necessary for forming the geopolymer matrix. The substantial increase in strength observed in samples from FGT ash dried after water extraction, compared to those synthesised immediately after washing, underscores the necessity of maintaining a sufficiently high NaOH concentration. Therefore, these findings emphasise the importance of optimising the NaOH concentration during geopolymer synthesis processes involving FGT ash. Ensuring an appropriate NaOH concentration facilitates effective dissolution of aluminium and silicon oxides, ultimately leading to improved material strength and performance.

Current knowledge indicates that calcium silicate hydrate (CHS phase) is the main binding phase for cementitious materials as well as alkali-activated slags [49]. In the case of geopolymers, the binding properties of these materials are attributed to the formation of a three-dimensional amorphous network of aluminosilicates [50,51]. Class F or low-calcium fly ash (LCFA) is typically used as the primary source of aluminosilicates; however, heat curing is required to complete the reaction and curing process and produce a strong composite. Moreover, calcium additives are often required to improve the strength of low-calcium geopolymers [52].

The strength results highlight the significant impact of the water leaching process on the geopolymer samples synthesised from FGT ash. Samples obtained immediately after leaching (FGTm), where water was still present, exhibited lower strength after 28 days compared to their strength at 7 days, with a twofold decrease noted. This behaviour is consistent with findings observed by Yip et al. [53], who studied geopolymer samples formed from metakaolin and blast furnace slag (GGBFS). They observed that excess water in less viscous gels could lead to internal pores and reduced strength if not consumed during the hydration process.

In contrast, samples synthesised from FGT ash that was leached and subsequently dried before synthesis (FGTmDRY) showed improved strength characteristics across all combinations with B, K, or L ashes. These samples showed significantly higher strengths after both 7 and 28 days compared to those synthesised from wet ash (FGTm).

The presence of calcium in geopolymers is beneficial due to its role in forming the calcium silicate hydrate (CSH) phase, which contributes to strength development. However, an excess of calcium can also detrimentally affect strength [52,53]. The concentration of the alkaline activator, typically sodium hydroxide (NaOH), is crucial as well. At extremely high pH levels (around 14), calcium may not effectively participate in forming the CSH phase, as confirmed by X-ray diffraction (XRD) data [54]. Instead, the enhancement in strength due to calcium can be attributed to the formation of silicate and polysialate networks within the geopolymer matrix [55,56]. Indeed, highly alkaline solutions can lead to the precipitation of calcium hydroxide, which accelerates the solidification of geopolymers, as observed in the synthesis of samples using dried FGT ash (FGTmDRY). The strength results obtained from three series of samples—based on FGT baseline, moist ash after water washing (FGTm), and dried ash (FGTmDRY)—mixed with three fly ashes (B, K, and L) demonstrate a dependence on the amounts of injected silicon and aluminium.

**Figure 2:**
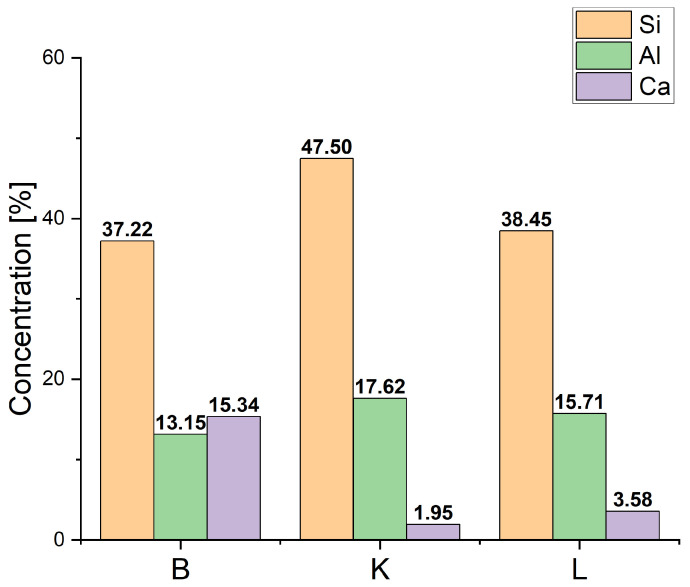
Percentage contents of silicon, aluminium, and calcium in B, K, and L fly ashes used in geopolymer synthesis.

**Figure 3:**
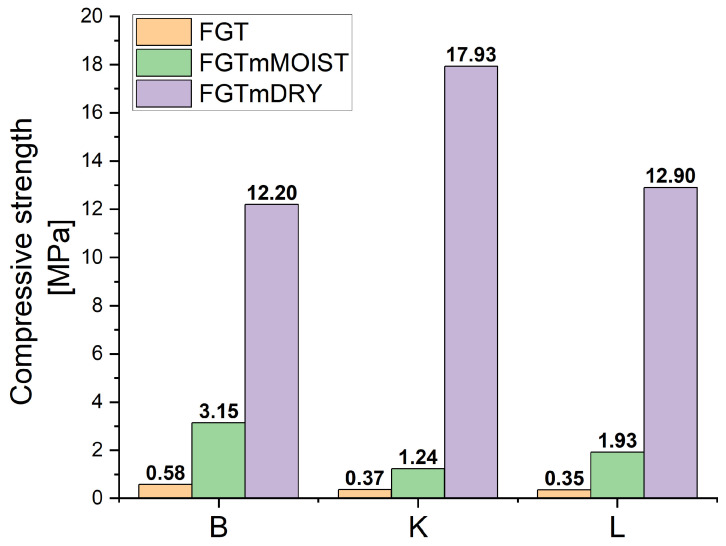
Overview of compressive strength of the obtained geopolymer samples after 28 days (explanation: FGT—initial ash, FGTm—moist FGT ash after water leaching, FGTmDRY—dried FGTm ash).

Figure 2 provides a quantitative overview of the dominant components in the fly ashes derived from coal combustion. Ash B, sourced from lignite coal, exhibits elevated calcium content. All three ashes—B, K, and L—share similar silicon/aluminium (Si/Al) ratios of approximately 2.8, 2.7, and 2.4, respectively. Comparing these data with Figure 3, which summarises the compressive strength results after 28 days for samples from the three test series, illustrates the significant impact of the water leaching process on FGT ash.

Furthermore, it is evident that the FGTmDRY+K sample exhibits the highest compressive strength. This outcome correlates with the higher silicon content introduced by the K ash, as well as the aluminium content that it contributes. This highlights the importance of optimising the composition and preparation of geopolymer materials to enhance their mechanical properties, taking into account the specific characteristics and ratios of silicon and aluminium from different fly ash sources.

### 5.2. Leachability of Heavy Metals

As a result of the ash extraction process using water, the aqueous filtrate and ash were analysed and later used for further syntheses.

The leaching process conducted with water (washing) facilitates the extraction of soluble elements from FGT ash, including metals like zinc and lead. The literature suggests using mineral acids (such as sulphuric acid or hydrochloric acid) or organic acids (like acetic acid or oxalic acid), based on their standard potential values for effective metal leaching [10]. While acids offer a chemical rationale for leaching, they also generate additional liquid waste, necessitating costly purification processes. Water, as a leaching agent in ash modification processes, is also under investigation. Research indicates that inorganic acids can leach up to 80% of zinc and lead, while organic acids range from 20% to nearly 50%. In comparison, water can solubilise approximately 13% of zinc [10]. The leaching of FGT ash carried out with water confirmed this relationship, as the zinc content in the aqueous solution after the leaching process was at a relatively high level.

**Figure 4:**
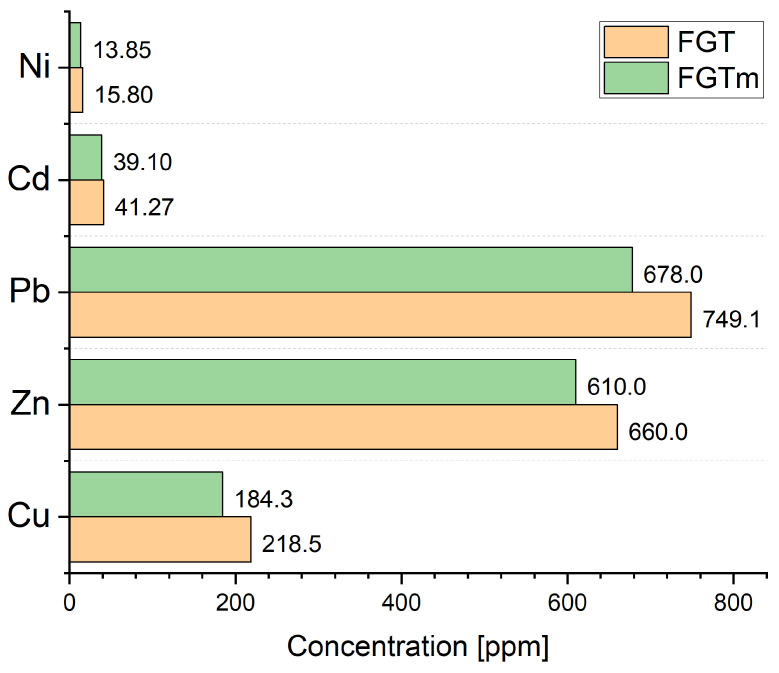
Contents of selected heavy metals in FGT ash before and after the leaching process with water.

Table 5 indicates high concentrations of potassium, sodium, and calcium, as well as relatively high amounts of zinc and aluminium. The removal of significant amounts of water-soluble calcium from FGT ash affected the compressive strength of samples obtained from ash after washing, supplemented with coal combustion ash (Table 2). In terms of the heavy metals contained in the ash, the leaching process practically did not change their proportions. Figure 4 demonstrates that the values before the leaching process were similar to those determined after.

Based on the strength tests conducted, two samples were chosen for subsequent leachability tests of selected heavy metals. The first sample selected for testing was FGTmodDRY+K, synthesised using dried FGT ash after the leaching process, with the addition of ash K. The second sample selected was a combination of moist ash after washing (FGTm) and commercial cement, synthesised at a volume ratio of 1:2.

Since the FGT ash used in the synthesis is categorised as hazardous waste, its inclusion in the new material necessitates verifying whether the hazardous components (heavy metals) have been immobilised within the geopolymer structure and do not exceed allowable standards.

**Figure 5:**
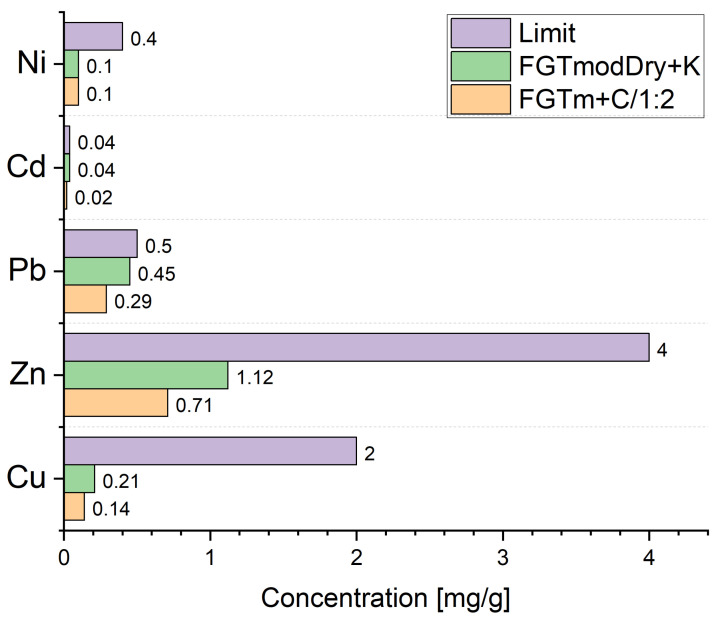
Heavy metal contents in leachate according to PB-10/BC (25 August 2009), based on the PN-EN ISO11885:2009 standard (the limit value in the figure refers to the Regulation of the Ministry of Economy of 16 July 2015 on admitting waste to storage at landfills).

A leachability analysis was performed for five heavy metals. Figure 5 presents the results, indicating that the material synthesised from FGTm ash with cement exhibited lower leachability of the tested heavy metals compared to the geopolymer sample obtained by adding K ash to the dried FGTm, especially for zinc and lead.

However, the results obtained for both samples met the standards outlined in the legislation of the European Union and Poland concerning leachate limit values for waste acceptable for landfills and classified as inert waste (Decision 2003/33/EC establishing criteria and procedures for acceptance of waste at landfills, pursuant to Art. 16 and Annex II to Directive 1999/31/EC, Regulation of the Ministry of Economy of 16 July 2015 on admitting waste to storage at landfills). These regulations specify the maximum permissible concentrations of copper, zinc, lead, cadmium, and nickel as 2, 4, 0.5, 0.04, and 0.4 mg/kg, respectively.

## 6. Conclusions

This research aimed to develop a methodology for producing geopolymer materials using FGT ash from dry flue gas desulphurisation in municipal waste incineration. Due to its high heavy metal contents, this ash is classified as hazardous waste, precluding landfill disposal. This study proposed a test cycle using both raw FGT ash and water-leached ash, supplemented with coal fly ash (from both lignite and coal) or commercial CEM I 42.5R cement to provide essential aluminium and silicon for geopolymer synthesis.

The key conclusions drawn from the research cycle are as follows:

1. Geopolymer synthesis using FGT ash and coal fly ash does not yield materials with sufficient strength parameters.
2. Washing FGT ash with water produces favourable results for subsequent geopolymer synthesis, especially when the ash is dried after the washing operation.
3. The materials produced met the leachability standards for the heavy metals tested in this study, qualifying them for disposal in landfills according to regulatory limits.

These results underscore the potential of water leaching to enhance the suitability of FGT ash for geopolymer applications while ensuring compliance with environmental standards for hazardous waste disposal.

## Figures and Tables

**Table 1:** The preparation of samples.

Sample Symbol	FGT	FGTm	FA	Cem	Sodium Silicate	Sodium Hydroxide 10 mol/dm^3^	Water
	cm^3^ g	cm^3^ g	cm^3^ g	cm^3^ g	g	g	g
FGT+B	100 60		200 170		80	40	100
FGT+K	100 60		200 170		80	40	100
FGT+L	100 60		200 230		80	40	100
FGTm+B		100 150	200 170		80	40	
FGTm+K		100 150	200 170		80	40	
FGTm+L		100 150	200 230		80	40	
FGTm+C(2:1)		100 150		200 250	120	60	
FGTm+C(1:2)		200 300		100 125	100	50	
FGTmDRY+B		95 (Dry) 100	200 170		80	40	
FGTmDRY+K		95 (Dry) 100	200 170		80	40	
FGTmDRY+L		95 (Dry) 100	200 230		80	40	

**Table 2:** Chemical composition of coal combustion fly ashes used in synthesis.

Compound	B Fly Ash	K Fly Ash	L Fly Ash
	Concentration, %		
Na_2_O	0.93	3.75	2.30
MgO	0.85	0.72	1.77
Al_2_O_3_	13.15	17.62	15.71
SiO_2_	37.22	47.50	38.45
P_2_O_5_	0.45	1.05	0.74
SO_3_	2.93	0.55	1.04
K_2_O	0.21	2.23	2.77
CaO	15.34	1.95	3.58
TiO_2_	1.59	1.32	1.00
V_2_O_5_	0.04	-	0.04
Cr_2_O_3_	0.02	0.03	0.05
MnO	0.02	-	0.17
Fe_2_O_3_	4.99	5.45	10.09
NiO	0.01	0.03	0.02
CuO	0.01	-	0.09
ZnO	0.01	0.06	0.99
BaO	0.02	0.06	0.09

**Table 3:** Example of heavy metal contents in ash from municipal waste incinerators.

Elements					References
Pb	Cd	Zn	Cu	Ni	
1350	129	15,600	707	100	[42]
5090	246	10,800	1270	N/A	[43]
4500	350	19,000	890	94	[8]
4600	500	22,000	98	61	[44]
1850	181	9322	500	38	[45]
1330	314	13,200	718	N/A	[13]
2075	255	7787	545	N/A	[46]

**Table 4:** Results of compressive strength tests for the prepared specimens.

Sample Symbol	Comprehensive Strength [MPa]	Comprehensive Strength [MPa]
	After 7 Days	After 28 Days
FGT+B	0.58	0.58
FGT+K	0.36	0.37
FGT+L	0.30	0.35
FGTm+B	6.10	3.15
FGTm+K	2.96	1.24
FGTm+L	3.16	1.93
FGTm+C(2:1)	1.60	2.01
FGTm+C(1:2)	8.51	11.12
FGTmDRY+B	7.88	12.20
FGTmDRY+K	17.70	17.93
FGTmDRY+L	11.79	12.90

**Table 5:** Selected results of aqueous solution analysis after FGT ash extraction with water.

Compound	Concentration	Result
Sb	mg/kg	40.0
Cr	mg/kg	3.80
Zn	mg/kg	1220
Al	mg/kg	1080
Cd	mg/kg	11.2
Si	mg/kg	248
Cu	mg/kg	28.1
Ni	mg/kg	<1.0
Pb	mg/kg	234
K	mg/kg	72,800
Na	mg/kg	157,000
Ca	mg/kg	157,000
Fe	mg/kg	170

## Data Availability

The original contributions presented in the study are included in the article, further inquiries can be directed to the corresponding author.
